# Her Heart's Mistake

**Published:** 1889-09-14

**Authors:** E. D. Cumming

**Affiliations:** Author of "An Ordeal by Silence"


### Her Heart's Mistake.

By E. D. Cumming, Author of "An Ordeal by Silence."

*(Continued from page 364.)*

### Chapter XXII.—A Year After.

Kate Bairdsley was walking slowly homewards along the Ridge-road, outside the walls of Delhi. The gentle slope on her left, covered with low jungle and huge boulders, stretched away down to orchards and native "bustees," or mud-built villages, and beyond them to the broad Jumna. It was hard to realise that this was the scene of the greatest struggle of the mutiny; that this rugged slope was once scarred with the British trenches, of which never a trace was visible now. Anglo-Indians think little of the deeds which the names of Delhi, Cawnpore, and Lucknow suggest to the minds of fellow-countrymen at home, but Kate could never take her evening walk along the Ridge without calling up a vision of the days gone by, when the old walls, now breached for road and railway, presented their unbroken front to the besiegers' guns. Thoughts like these are generally left for the minds of cold weather pleasure-seekers from England; to the resident in the country, Delhi is a charming station, where shooting and polo are to be had at their best; while Cawnpore and Lucknow are the pig-stickers' Elysian fields. The "Well" and the "Residency" are monumental accidents which the new comer goes to visit as a matter of duty on his first arrival, and passes by ever afterwards.

Kate had changed greatly since we last saw her at Meerut; a sad, listless expression had replaced the old vivacity, and she was thinner, older, and more womanly. Twelve months had much to answer for in its treatment of her beauty.

"Well, have you been moping about the Ridge as usual?" asked her husband, as she entered the verandah.

"Yes, I went for a stroll there."

"You're growing precious fond of your own society. I hope you find it more exhilarating than I do."

Kate did not reply, but disappeared into her own room. She was used to George's sneers now. His affectionate manner had soon become one of indifference, then petulant, and in course of time harshly neglectful. He never attempted to seek her companionship, nor did he affect the slightest interest in her pursuits. The matrimonial tie was a handcuff to both.

"Why on earth can't you try and look a little more cheerful?" he would ask roughly, irritated by her depression and silence. "Anyone would think I was in the habit of beating you. Do put on a slightly more amiable expression. It's enough to give a fellow the blues to look at you."

But taunts and hectoring were equally ineffectual, though neither failed for lack of trial. Bairdsley fretfully told himself that he didn't know what had come over her. She was sulky and stupid during their honeymoon at Simla, and he had had much ado to be civil to her then. But since the regiment had come to Delhi, her sullenness had grown intolerable. She never spoke, she moped in her room alone, drove alone and walked alone, morning and evening; he had done his best to shake her out of this troublesome mood, but it was quite useless; she would not wake up and behave like a reasonable being. He had given up bothering himself about her long ago, and sought amusement at the club and the polo ground whither, she seldom or never accompanied him. He dined at mess more often than not, and her indifference to his neglect piqued him more than the loudest complaints could have done. Their home was comfortless and unhappy, for Bairdsley's ungovernable temper had not improved, and he vented it upon his domestics so unsparingly that his wife's kindlier management was outweighed. They could not get decent servants though they paid the same wages as everybody else, and cook after cook had been tried and dismissed; sometimes under chastisement, but sometimes scatheless, had he only time to get clear of the cookhouse before "master" entered the narrow door.

"Is there nothing to eat but this everlasting goat and moorghi;" he would ask savagely when the same dinner of spurious mutton and stringy fowl was served up night after night.

"You thrashed the last bobachee till he ran away," Kate would answer; "the matey cooked this, and I don't suppose he can do any better."

"Then the sooner you get another cook the better."

Delhi bazaar had offered its scores in turn, and now no servant who could get a job elsewhere would put foot inside the Bairdsley's compound. Dr. Harrison had gone home soon after his daughter's marriage, and his entire establishment had come to her when he went, but they left one after another, kissing the feet of her who to them was still the "Missie Baba," and cursing the mother of the Captain Sahib who bare him. They could not put up with his brutality even for her sake, and they vanished and scattered over the face of the earth to seek masters who knew how to treat them properly.

"For the future, please make the dinner-hour seven o'clock," said Bairdsley, one evening after the fourth new cook had taken flight, "so that if there's nothing fit for me to eat I shall have time to change and go up to the mess."

And Kate received his order, as she did his taunts, in silence, and with secret thankfulness made the alteration. She often had the evenings to herself afterwards, for he did not always wait to see what the dinner was like before he made up his mind to go to the fort. This arrangement had been made six months ago, and she had grown accustomed to it.

She had incurred her father's lasting displeasure by her apparent vacillation and weak-mindedness. He had come to see her on his way home, and, to her intense relief, they had become reconciled; she took up a heavy burden when she married Bairdsley, and her father's anger had added much to its weight. She had never seen Richard Warren since that night when she consented to marry him, nor had she heard of him, save indirectly. He had had brain fever after her marriage, and had been sent to England when he recovered, but where he was now she did not know. He must have passed through Delhi on his way from Meerut to Bombay, but had not let her know anything of his presence. She had written to him once—during that miserable honeymoon she spent at Simla—telling him how she had been coerced into fulfilment of her promise, and begging his forgiveness. A passionate, incoherent letter it had been, but he never answered it; and now that she had long given up hope of hearing from him she told herself that it was well. Though she did not believe that Bairdsley would care whether she corresponded with his rival or not, she was anxious to do her duty as a wife, hard and thankless as she found the task.

On this particular evening her husband was more surly and morose than usual; he had lamed his best polo pony that afternoon, and he had received some letters from home whose contents he did not confide to Kate, but which appeared to give him much uneasiness.

"You'll have to give up your plans for going to the hills next April," he said, brusquely, as they sat down to dinner. The suggestion that she should spend the next hot season at Simla had been his own, as Kate recollected, but she did not remind him of the fact.

"I don't care much about going to live alone in an hotel," she answered, "it's no great deprivation to remain here."

"Well, at any rate, I can't afford it, so you can make up your mind to stay in the plains."

"I hope nothing has gone wrong in money matters," she said, after a pause.

"Very good of you to say so, I'm sure," snarled Bairdsley.

It had been his avowed intention to retire from the service after his marriage and return to England, but for some reason with which Kate had not been made acquainted the plan had been indefinitely postponed. He had become more acclimatised, and though he could not take liberties with his health, he was quite able to join in most sports without fear of prostration by the heat. The tribal disturbances on the North-West frontier had assumed larger proportions latterly, and the 110th had been mentioned as one of the British regiments likely to be first sent to the front in case of need.

"Have you heard from your father lately?" enquired Bairdsley, after a silence of ten minutes, which had only been broken by his maledictory remarks to the khitmugar.

"I heard this morning, as usual," replied Kate.

"Had he any news?"

"None in particular. He is going to take a small house at Cheltenham, as he has been staying there for a few months and likes the place; but I'll give you his letter after dinner."

He said no more then, but asked her to bring it to him when they left the table. Kate was a little surprised at his unusual interest in her correspondence, but soon forgot the incident when he read and returned the letter without comment upon its contents. She never thought of connecting his curiosity regarding her father's letter with the uneasiness he had betrayed that morning on perusing one he had received himself from a correspondent whose handwriting was unknown to her. Nor did her frank, open nature suggest that he had any ulterior motive, when on each succeeding mail day after that he asked to see Dr. Harrison's letters. Only once he made any remark upon his father-in-law's news. The Doctor was complaining of the rigours of the English winter, and said that he was contemplating a change to Cannes or Nice for a few months.

"That's an extraordinary notion of your father's," he said, as he gave Kate the letter.

"What is?"

"The idea of wintering in the Riviera."

"I don't see anything very remarkable about it; on the contrary, I think it's the most natural thing for him to do."

"Do you think he will do it?" asked Bairdsley.

"Very possibly," answered Kate, wondering what had brought that anxious look to her husband's face. "Why should he not?"

"Of course there's no reason why he shouldn't; but I spent January at Mentone once, and it was perfectly abominable. Two-thirds of the people lived in Bath chairs, and the rest had muzzles on."

"Papa's speciality is diseases of the chest; so he might find some professional work there to amuse himself with. He hates being idle."

Kate often asked herself afterwards what there had been in this innocent observation to make Bairdsley bounce out of his chair and tell her she was talking infernal nonsense. She got an explanatory answer in the course of time; but she observed that next incoming mail day he looked eagerly for the letters, and asked to see the Doctor's as soon she had read it.

The delightful Indian cold season was at its best when Kate's son was born. That bright, cold January morning was the beginning of a new life to her. She had something to live for now, and her husband became more kindly and good-humoured in his manner towards her.

"We must try and get you off to Simla before the hot weather," he said one evening, as he looked at his heir slumbering in the arms of the soldier's wife who was acting as nurse.

Kate felt no pang at the thought that he would willingly incur for the child an expense which he had grudged upon her own account. She was nothing to him, and knew it, but the week-old boy was heir to a fine estate, and his father must naturally take an interest in him.

Old Mr. Bairdsley wrote occasionally to her, and she had conceived a warm affection for her father-in-law, albeit she only knew him through his letters. News of the great event had been telegraphed both to him and to Dr. Harrison, and since its occurrence Kate's thoughts had dwelt much upon her future return to England, though there seemed to be little prospect of it for at least three years, when the regiment expected to be ordered home.

She longed to return. Although she never dared confess it to herself, her love for Warren was as true and deep as it had been a year ago. Though they might live within call of each other she knew that she would be safe with him, even under George's jealous eye, for Warren's sense of honour was as sound as her own, and better balanced. Her father had mentioned his name once, some months ago, before Bairdsley had commenced the practice of reading his letters. He had been up in town for a day or two, and having encountered the barrister in the street, had taken him to his hotel, where they spent a long evening together.

"He has changed a good deal since his illness," wrote the Doctor, "but otherwise is just the same thoughtful, pleasant companion we knew in Meerut. He has been more fortunate in his profession since his return, and tells me that he has a very satisfactory practice, though his old nervous shyness hampers his advance sadly. He is coming to spend a week or ten days at Cheltenham with me when I settle down."

The letter did not say that Warren had made any enquiry about her, nor did he appear to have sent her the most trivial message; and for a time Kate wondered whether his was one of those short memories which cannot last a year. The thought that she had been so soon forgotten pained her; but she told herself that she deserved nothing else, and that it was her duty to forget Warren, too, now she was Bairdsley's wife.

